# Comparative chloroplast genome analyses of diverse *Phoebe* (Lauraceae) species endemic to China provide insight into their phylogeographical origin

**DOI:** 10.7717/peerj.14573

**Published:** 2023-02-03

**Authors:** Wenbo Shi, Weicai Song, Zimeng Chen, Haohong Cai, Qin Gong, Jin Liu, Chao Shi, Shuo Wang

**Affiliations:** 1 College of Marine Science and Biological Engineering, Qingdao University of Science and Technology, Qingdao, China; 2 Yunnan Institute of Tropical Crops, Xishuangbanna, China; 3 Plant Germplasm and Genomics Center, Germplasm Bank of Wild Species in Southwest China, Kunming Institute of Botany, The Chinese Academy of Sciences, Kunming, China

**Keywords:** *Phoebe*, Chloroplast genome, Comparative analysis, Phylogeny, Phylogeographical origin, Conservation

## Abstract

The genus *Phoebe* (Lauraceae) includes about 90 evergreen tree species that are an ideal source of timber. Habitat destruction and deforestation have resulted in most of them being endemic to China. The accurate identification of endangered *Phoebe* species in China is necessary for their conservation. Chloroplast genome sequences can play an important role in species identification. In this study, comparative chloroplast genome analyses were conducted on diverse *Phoebe* species that are primarily distributed in China. Despite the conserved nature of chloroplast genomes, we detected some highly divergent intergenic regions (*petA–psbE*, *ndhF–rpl32*, and *psbM–trnD*-GUC) as well as three highly divergent genes (*rbcL*, *ycf1*, and *ycf2*) that have potential applications in phylogenetics and evolutionary analysis. The phylogenetic analysis indicated that various *Phoebe* species in China were divided into three clades. The complete chloroplast genome was better suited for phylogenetic analysis of *Phoebe* species. In addition, based on the phylogeographical analysis of *Phoebe* species in China, we inferred that the *Phoebe* species in China first originated in Yunnan and then spread to other southern areas of the Yangtze River. The results of this research will add to existing case studies on the phylogenetic analysis of *Phoebe* species and have the potential to contribute to the conservation of *Phoebe* species that are in danger of extinction.

## Introduction

The genus *Phoebe* (Lauraceae) includes nearly 90 species primarily distributed in Asia (Song et al., 2017a), and about 34 species and three varieties are endemic to China and occur in the Yangtze River Valley and its surrounding southern areas (Chen et al., 2020b). In China, *Phoebe* wood is known for its excellent material for use in fine architecture, furniture, and carvings (Li et al., 2017). Owing to habitat destruction and deforestation, the majority of surviving populations consist of fewer than 70 individuals (Chen et al., 2020a; Xiao et al., 2020). Over the years, cultivating, developing, utilizing, and conserving these valuable tree species have been difficult (Gao et al., 2016). It is now more crucial than ever to protect *Phoebe* species in China. Before creating conservation and management strategies, the appropriate management authorities must correctly identify the species (Liu et al., 2018; Liu et al., 2021). Traditional taxonomy requires a detailed analysis of the morphological characteristics of the species, which necessitates accurate identification by taxonomists. When identifying large sample sizes or morphologically conservative taxa, traditional taxonomy is vulnerable to human error (Sheth & Thaker, 2017). The complete chloroplast genome could be used in species identification as an adjunct to traditional taxonomy (Wu et al., 2021).

Chloroplast genome sequences can provide important genetic information for conservation studies (Deng et al., 2019). A rising amount of research points to the complete chloroplast genome as a useful tool for learning about the phylogenetic and evolutionary background of plants (Song et al., 2022a; Song et al., 2022c). With the development of high-throughput sequencing technology, a large number of chloroplast genome sequences have been deposited in the National Center for Biotechnology Information (NCBI) databases, which provide important source data for chloroplast genome studies (Chen et al., 2017). Although chloroplast genomes are relatively conserved in structure, they are also subject to gene loss, mutation, and pseudogenization (Henriquez et al., 2020). These divergences can be employed for species identification and phylogenetic analysis to enhance the present understanding of the phylogenetic and evolutionary relationships of plants (Song et al., 2017b). The complete chloroplast genome sequence, in contrast to variable markers, is rich in genetic variation and has a potent ability to distinguish between closely related species (Chen et al., 2018; Krawczyk et al., 2018; Jiao et al., 2019).

Interest in biogeographic study has developed as a result of the current biodiversity problem and the urgent need to manage biodiversity (Schluter & Pennell, 2017; Médail & Baumel, 2018). In the recent years, a number of research have surfaced that analyze and comprehend plant diversity using biogeographic methodologies and then use those methods to conserve related species (Médail & Baumel, 2018; Bobo-Pinilla et al., 2022). In addition, it has been found that phylogeography is a useful tool for obtaining an accurate representation of the history and evolution of a species (Yang et al., 2019; Wang et al., 2021c). Chloroplast genomes have been used in certain research for phylogeography (Zhao, Pan & Zhang, 2019; Wang et al., 2021c). A more effective conservation approach for the species in question can be provided by tracing the origin and dispersal of species, particularly rare, endangered, and endemic plants (Duan et al., 2020; Bobo-Pinilla et al., 2022).

To date, phylogenetic analyses of *Phoebe* taxa in China are only based on highly variable markers. In the meanwhile, the published studies on *Phoebe* species have not yet performed phylogenetic analyses based on the complete chloroplast genome, and the comparative analyses of the species were rare. Additionally, the origin of *Phoebe* species in China has not been identified (Li et al., 2017; Song et al., 2017a). The aims of the present study were: (a) to characterize the chloroplast genomes of various *Phoebe* species in China; (b) to explore the divergences in the chloroplast genomes of various *Phoebe* species in China and identify highly variable regions; and (c) to reconstruct the phylogenetic relationships of various *Phoebe* species in China and to trace their phylogeographical origin. To determine the characteristics and divergence of the genus *Phoebe* in China, we chose nine *Phoebe* species for comparative analysis in accordance with the geographical differentiation and economic relevance of *Phoebe* species. Among these species, *P. hunanensis* is a representative *Phoebe* species native to Hunan Province and the Yangtze River Basin (Yang et al., 2022). *P. tavoyana* and *P. puwenensis* are valuable tropical timber trees found primarily in Yunnan province (Omar et al., 2020; Zhang, Li & Wang, 2020). Southwestern China is the primary distribution region for *P. neurantha*, *P. omeiensis*, and *P. zhennan* (Song et al., 2017a; Xiao et al., 2020; Wang et al., 2021a). In southeastern China, *P. bournei*, *P. chekiangensis*, and *P. sheareri* are all widely distributed (Li et al., 2017; Song et al., 2017a). Due to their excellent wood material, these *Phoebe* species all have significant economic significance. To investigate the phylogenetic relationships of the genus *Phoebe*, we constructed phylogenetic trees using all *Phoebe* species from the NCBI database.

## Materials & Methods

### Sample collections, DNA extraction from samples, and sequencing

We collected samples of two *Phoebe* species from the main area of their distribution. The fresh leaves of *P. hunanensis* were collected in Xiangxi Prefecture, Hunan Province, China (27°43′N, 110°22′E). Some young leaf blades of *P. hunanensis* were reddish purple adaxially. The fresh leaves of *P. tavoyana* were collected in Kunming City, Yunnan Province, China (24°23′N, 102°10′E). The leaf blades of *P. tavoyana* were lanceolate or elliptic-lanceolate, with a caudate-acuminate apex. The interval distance for proper specimen collection was taken into account when collecting each of the same species. Five representative individuals were selected for each species. All samples were identified by the author, Prof. Chao Shi. All plant materials were collected in accordance with all current laws and regulations, and the collection was approved by the local government. The voucher samples were stored at Qingdao University of Science and Technology (Chao Shi, chsh1111@aliyun.com) under the specimen codes PL202114 and PL202115. A modified high salt method previously reported (Shi et al., 2012) was used to extract chloroplast DNA from about 30 g of fresh mature leaf samples of two *Phoebe* species. Both the quantity and quality of the extracted DNA were assessed by spectrophotometry, while the integrity was assessed by 1% (*w*/*v*) agarose gel electrophoresis (Green & Sambrook, 2019). DNA of high quality was delivered to Novogene (Beijing, China) for genomic library construction and sequencing with the Illumina HiSeq platform (Illumina, San Diego, CA, USA). About 4.8 Gb of high quality, 2 × 150 bp paired-end raw reads were obtained and were used to assemble the complete chloroplast genomes of these two *Phoebe* species.

### Chloroplast genome *de novo* assembly and annotation

We used Trimmomatic v0.39 software (Bolger, Lohse & Usadel, 2014) to preprocess the acquired raw data, which included removing adapter sequences and additional sequences that were introduced during the sequencing process, eliminating low quality reads and reads with long stretches of N bases, and so on. FASTQC v0.11.9 (Brown, Pirrung & Mccue, 2017) and MULTIQC v1.12 software (Ewels et al., 2016) were used to assess the quality of newly produced clean short reads. The data were screened to retain reads of high quality with mean Phred scores greater than 35. Chloroplast-like (cp) reads were separated from clean reads by BLAST v2.6.0 (Johnson et al., 2008) based on comparison with a reference genome (*Phoebe neurantha*, NC_039620). Assembled short reads were *de novo* spliced into long contigs using SOAPdenovo v2.04 (Luo et al., 2012) with k-mer values set to 35, 44, 71, and 101. Geneious v 8.1 (Kearse et al., 2012) was used to complete the long-contigs sequence expansion and for gap filling. Then, tRNAscan-SE v1.21 (Schattner, Brooks & Lowe, 2005) was used to detect tRNA genes with the default settings, and RNAmmer v.1.2 (Lagesen et al., 2007) was used to validate rRNA genes again using the default settings. The chloroplast genome was annotated by GeSeq v1.42 (Tillich et al., 2017), while Sequin v16.0 (Lehwark & Greiner, 2018) was used to manually correct codons and gene boundaries. The circular chloroplast genomic map of *Phoebe* was drawn using Chloroplot v0.2.4 (Zheng et al., 2020). The two *de novo* assembled *Phoebe* chloroplast genomes were deposited in GenBank under accession numbers MZ442606 and MZ442607.

### Chloroplast genome structural analysis

The perl script MISA v2.1 (Beier et al., 2017) was applied to detect chloroplast genomic simple sequence repeats (SSRs), and the basic repeat settings for SSRs were determined to be nine for mononucleotides, four for dinucleotides, and three for trinucleotides, tetranucleotides, pentanucleotides, and hexanucleotides. The REPuter v2 (Kurtz et al., 2001) was employed to analyze forward (F), palindromic (P), reverse (R), and complement (C) repeats with a minimum repeat size of 20 bp and a maximum repeat size of 300 bp. Relative synonymous codon usage (RSCU) and codon usage frequency in the sequences of protein-coding genes were calculated by MEGA-X (Kumar et al., 2018). The heat map of the RSCU analysis was created using the R package of the heatmap. MAFFT v725 was applied to the protein-coding genes. The non-synonymous substitution rate (Ka)/ synonymous substitution rate (Ks) values of each *Phoebe* species were calculated with *P. neurantha* (NC_039620) as a reference sequence using KaKs_Calculator v2 (Wang et al., 2010).

### Genome comparison

The basic features of the chloroplast genomes of *Phoebe* species were compared and analyzed with Geneious v8.1, including the measurement of sequence length in each region, the proportion of different sequences, and the GC content of various regions. The comparative analysis of the whole sequence identity of the chloroplast genomes was performed using mVISTA v2.4 (Frazer et al., 2004) with the chloroplast genome of *P. neurantha* (NC_039620) as the reference sequence. The genes of the IR/single copy (SC) boundaries in the chloroplast genomes of the *Phoebe* species were compared and visually represented using IRscope v1.1 (Amiryousefi, Hyvönen & Poczai, 2018) to reveal contraction and expansion of the IR regions. After sequence alignment using MAFFT v725 (Katoh & Standley, 2013), the single nucleotide polymorphism (SNP) and insertion/deletion (InDel) variants were identified using DnaSP v6 software (Rozas et al., 2017). The sliding window was set to 800 bp, and the step size to 200 bp.

### Phylogenetic analysis

A phylogenetic analysis was conducted based on chloroplast genomes from 24 Lauraceae species, including those of the two *Phoebe* species sequenced and assembled in this study and another 22 downloaded from GenBank (Table S1). We used *Litsea pungens* and *Sinopora hongkongensis* as outgroups, together with 22 species of *Phoebe* to construct the phylogenetic trees. Maximum likelihood (ML) and Bayesian inference (BI) approaches were used for phylogenetic analysis. To create sequence alignments for the construction of phylogenetic trees, MAFFT v725 was applied to the complete cp genome sequence data. While MACSE v2 (Ranwez et al., 2018), which is designed for codons, was applied to a separate dataset of 79 protein-coding genes. Gblocks v0.91b (Talavera & Castresana, 2007) was then used to deal with the aligned sequences. The generalized-time reversible (GTR) with invariants (I) and discrete Gamma (G) (GTR+I+G) model was identified as the best fitting substitution model by applying the Bayesian information criterion (BIC) using jmodeltest v2.1.10 (Darriba et al., 2012). Lastly, phylogenetic trees, with branch support based on 1,000 bootstrap replicates, were inferred using the ML method as implemented in MEGA-X software (Kumar et al., 2018). MrBayes v3.2.7 (Huelsenbeck & Ronquist, 2001) was used for the BI analysis. The Markov chain Monte Carlo (MCMC) method was run for 2,000,000 generations. Each 5,000 generations, samples of trees were taken. The consensus tree was created using the remaining trees after the first 25% of the trees were regarded as “burn-in” and discarded. The GTR+I+G model of evolution was applied to both MEGA-X and MrBayes v3.2.7.

### Ancestral geographical area reconstruction

We performed an ancestral geographical area reconstruction to trace the biogeographic history of the *Phoebe* species in China. Based on the distribution of 22 *Phoebe* species in China as recorded in the literature and in botanical specimens, we have produced biogeographic data on these species (Table S1). We used the ancestor reconstruction software RASP v4 (Yu, Blair & He, 2020) to forecast the origin and dispersal of *Phoebe* species in China utilizing S-DIVA (Statistical Dispersal-Vicariance Analysis) and BBM (Bayesian Binary MCMC) methodologies. For the reconstruction of the ancestral geographic area, the ML tree of the complete chloroplast genome from the phylogenetic analysis was employed, with the BI tree of the complete chloroplast genome serving as a supplement. At each node, there were a maximum of four areas. The S-DIVA study employed 1,000 randomly chosen trees out of 4,000 trees, with all parameters set to their default settings. In the BBM analysis, 10 MCMC chains were applied to run using the Jukes-Cantor (JK) model, with all settings left at their default levels.

## Results

### Chloroplast genome features of *Phoebe*

**Figure 1:**
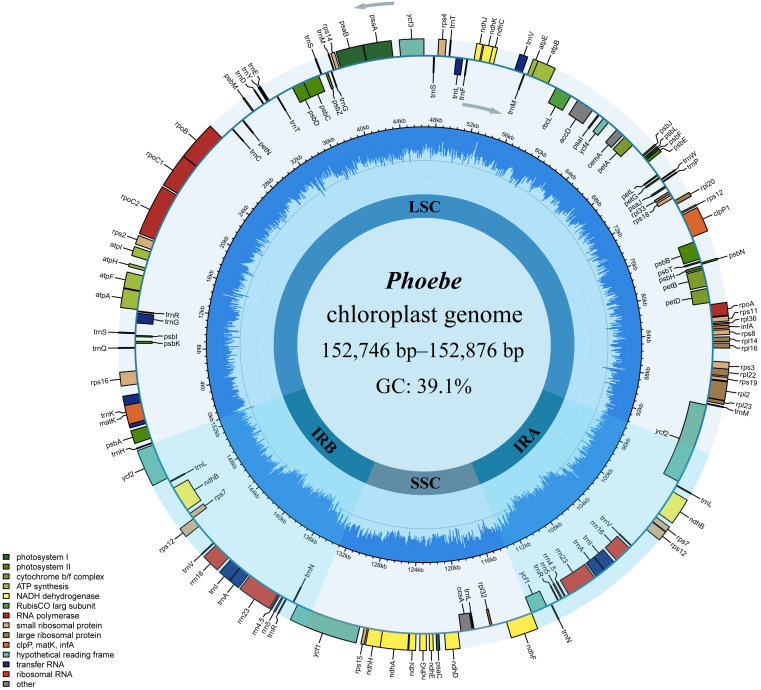
The circular map of *Phoebe* chloroplast genomes. The genes are shown along the inner and outer sides of the circle, with those on the outside of the circle transcribed counterclockwise and those on the inside of the circle transcribed clockwise. Genes with different functions are shown in different colors, as shown in the bottom left corner. The inner circles are divided into dark blue and light blue to indicate GC and AT content, respectively. The large single copy (LSC), the small single copy (SSC), and the inverted repeat (IRA and IRB) regions are also represented.

The comparative chloroplast genome analysis of nine representative species in the genus *Phoebe* (*P. hunanensis, P. tavoyana*, *P. bournei*, *P. chekiangensis*, *P. neurantha*, *P. omeiensis*, *P. puwenensis*, *P. sheareri*, *P. zhennan*) was used to assess the genome features of *Phoebe* (Table S1). The chloroplast genome sizes of the *Phoebe* species ranged from 152,746 bp for *P. puwenensis* to 152,876 bp for *P. sheareri* (Table 1). As observed in the majority of other angiosperms, all nine species of *Phoebe* presented a classical quadripartite structure (Fig. 1) (Wicke et al., 2011). The length of the large single copy (LSC) region ranged from 93,685 bp to 93,966 bp. The small single copy (SSC) region varied from 18,899 bp to 18,967 bp in length, and that of the IR regions ranged from 19,980 bp to 20,076 bp. The protein-coding regions of *P. hunanensis* accounted for 49.01% of its complete chloroplast genome, while rRNA and tRNA, introns, intergenic sequences, and pseudogenes represented 7.57%, 12.29%, and 30.95%, respectively. The respective proportions of *P. tavoyana* were quite consistent with those of *P. hunanensis*. The total GC content of the chloroplast genome was equally high (39.1%) (Table 1), with the IR regions having the highest GC content (44.4%), followed by the LSC (38.0%) and SSC (33.8%) regions. A total of 128 genes were identified, consisting of 84 protein-coding genes, 36 tRNA genes, and 8 rRNA genes (Table 2). Among the protein-coding genes, nine genes (*atpF, petB, petD, ndhA, ndhB, rpoC1, rps12, rps16,* and *rpl2*) contained a single intron, two genes (*ycf3* and *clpP*) possessed two introns, and six tRNA genes (*trnA*-UGC, *trnG*-UCC, *trnI*-GAU, *trnL*-UAA, *trnK*-UUU, and *trnV*-UAC) featured a single intron (Table 2).

**Table 1:** Chloroplast genome features of *Phoebe* species.

**Genome features**	*Phoebe hunanensis*	*Phoebe tavoyana*	*Phoebe bournei*	*Phoebe chekiangensis*	*Phoebe neurantha*	*Phoebe omeiensis*	*Phoebe puwenensis*	*Phoebe sheareri*	*Phoebe zhennan*
Genome size (bp)	152,791	152,814	152,853	152,849	152,782	152,855	152,746	152,876	152,831
LSC size (bp)	93,713	93,769	93,777	93,772	93,731	93,966	93,685	93,893	93,753
SSC size (bp)	18,928	18,899	18,928	18,967	18,905	18,929	18,909	18,915	18,928
IR size (bp)	20,075	20,073	20,074	20,055	20,074	19,980	20,076	20,034	20,075
Total GC content (%)	39.1%	39.2%	39.1%	39.1%	39.2%	39.1%	39.1%	39.1%	39.1%
GC content in LSC (%)	38.0%	37.9%	38.0%	38.0%	38.0%	37.9%	37.9%	37.9%	38.0%
GC content in SSC (%)	33.8%	34.0%	33.9%	33.8%	33.9%	33.8%	33.9%	33.9%	33.9%
GC content in IR (%)	44.4%	44.4%	44.4%	44.4%	44.4%	44.5%	44.4%	44.5%	44.4%
Number of genes (unique)	128(111)	129(112)	127(111)	127(111)	126(113)	127(111)	127(113)	127(111)	127(111)
Protein genes (unique)	84(79)	85(79)	81(79)	81(79)	82(79)	81(79)	82(79)	81(79)	81(79)
tRNA genes (unique)	36(29)	36(29)	36(29)	36(29)	36(29)	36(29)	36(29)	36(29)	36(29)
rRNA genes (unique)	8(4)	8(4)	8(4)	8(4)	8(4)	8(4)	8(4)	8(4)	8(4)
Protein-coding regions (%)	49.01%	49.44%	46.16%	46.16%	46.52%	46.16%	46.70%	46.15%	46.16%
rRNA and tRNA (%)	7.57%	7.71%	7.71%	7.70%	7.44%	7.70%	7.70%	7.70%	7.70%
Introns size (% total)	12.29%	12.29%	15.81%	15.81%	15.83%	15.80%	16.21%	15.26%	15.82%
Intergenic sequences and pseudogenes (%)	30.95%	29.90%	30.27%	30.26%	32.84%	30.30%	30.25%	30.30%	30.25%

**Table 2:** List of predicted genes in the *Phoebe* chloroplast genomes.

Photosynthesis related genes	Large subunit of rubisco	*rbcL*
	Photosystem I	*psaA, psaB, psaC, psaI, psaJ*
	Assembly/stability of photosystem I	ycf3^**^, ycf4
	Photosystem II	*psbA, psbB, psbC, psbD, psbE, psbF, psbH, psbI, psbJ, psbK, psbL, psbM, psbN, psbT, psbZ*
	ATP synthase	atpA, atpB, atpE, atpF^*^, atpH, atpI
	Cytochrome b6/f complex	petA, petB^*^, petD^*^, petG, petL, petN
	Cytochrome c synthesis	*ccsA*
	NADH dehydrogenase	ndhA^*^, ndhB^*^, ndhC, ndhD, ndhE, ndhF, ndhG, ndhH, ndhI, ndhJ, ndhK
Transcription and translation related genes	RNA polymerase subunits / transcription	rpoA, rpoB, rpoC1^*^, rpoC2
	Small subunit of ribosomal proteins	rps11, rps12^*^ ^,^ ^a^ , rps14, rps15, rps16^*^, rps18, rps19, rps2, rps3, rps4, rps7 ^a^ *, rps8*
	Large subunit of ribosomal proteins	rpl14, rpl16, rpl2^*^, rpl20, rpl22, rpl23 ^a^ *, rpl32, rpl33, rpl36*
	translation initiation factor	*infA*
RNA genes	ribosomal RNA	*rrn16* ^a^ *, rrn23* ^a^ *, rrn4.5* ^a^ *, rrn5* ^a^
	transfer RNA	*trnA-* UGC^*^^,^^a^*, trnR-* ACG^a^*, trnR-* UCU*, trnN-* GUU^a^*, trnD-* GUC*, trnC-* GCA*, trnQ-* UUG*, trnE-* UUC*, trnG-* GCC*, trnG-* UCC^*^, trnH- GUG*, trnI-* GAU^*^^,^^a^*, trnL-* CAA^a^*, trnL-* UAA^*^, trnL- UAG*, trnK-* UUU^*^, trnfM- CAU*, trnM-* CAU^a^*, trnF-* GAA*, trnP-* UGG*, trnS-* GCU*, trnS-* GGA*, trnS-* UGA*, trnT-* GGU*, trnT-* UGU*, trnW-* CCA*, trnY-* GUA*, trnV-* GAC^a^*, trnV-* UAC^*^
Other genes	*RNA processing*	*matK*
	*carbon metabolism*	*cemA*
	*fatty acid synthesis*	*accD*
	*proteolysis*	clpP^**^
	*component of TIC complex*	*ycf1* ^a^
	*hypothetical proteins*	*ycf2* ^a^

**Notes.**

* Gene with one intron.

** Gene with two introns.

a Gene with two copies.

### SSR and long repeat identification

**Figure 2:**
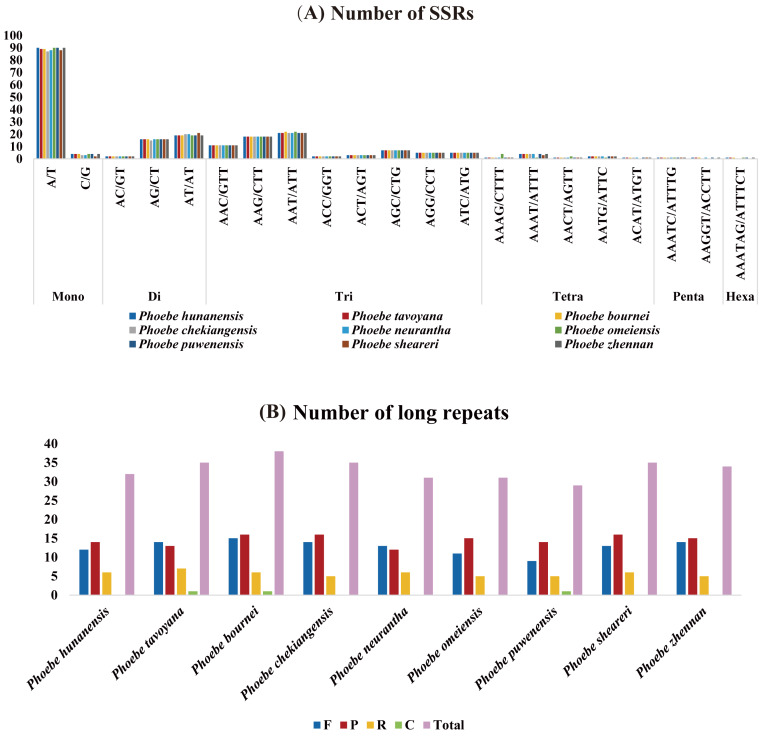
The analysis of short simple repeats (SSRs) and long repeats in *Phoebe* chloroplast genomes. (A) Number of SSRs. (B) Number of long repeats.

The distribution of SSRs in the chloroplast genomes of *Phoebe* species was analyzed using MISA v2.1. The quantity of SSRs ranged from 209 (*P. neurantha*) to 215 (*P. hunanensis*), and all species had similar distributions of SSRs types (Fig. 2A). There were 94 mononucleotides, 37 dinucleotides, 72 trinucleotides, nine tetranucleotides, two pentanucleotides, and one hexanucleotide in *P. hunanensis*. Mononucleotide repeats were the most abundant, comprising almost 43.41% of the total SSRs, followed by trinucleotide (33.87%), dinucleotide (17.51%), and tetranucleotide (4.12%) repeats. Pentanucleotide repeats (0.78%) and hexanucleotide repeats (0.31%) were extremely rare in these chloroplast genomes. The mononucleotide SSRs were most often comprised of A/T repeats (about 95.74% of all mononucleotide SSRs) (Table S2).

The long repeat sequences in nine *Phoebe* chloroplast genomes were analyzed. We found forward (F), palindromic (P), reverse (R), and complement (C) repeats of each repeat unit of at least 20 bp (Table S3). There were 29 (*P. puwenensis*) to 38 (*P. bournei*) long repeat sequences in each *Phoebe* chloroplast genome analyzed. Reverse and complement repeats were significantly less common than forward and palindromic repeats. The palindromic repeats were most abundant, except for *P. tavoyana* and *P. neurantha*, which had more forward repeats than palindromic repeats. Complement repeats were extraordinarily rare in the chloroplast genomes, occurring only once in *P. tavoyana*, *P. bournei*, and *P. puwenensis* (Fig. 2B). We measured the long repeats of lengths from 20 to 300 bp and found that long repeats of 20–21 bp in length were the most common (Table S3). We analyzed the region of repeats and found that the long repeat sequences were mainly distributed in the LSC region, followed by the IR and then SSC regions (Table S3).

### Codon preference analysis and estimation of evolutionary rates

**Figure 3:**
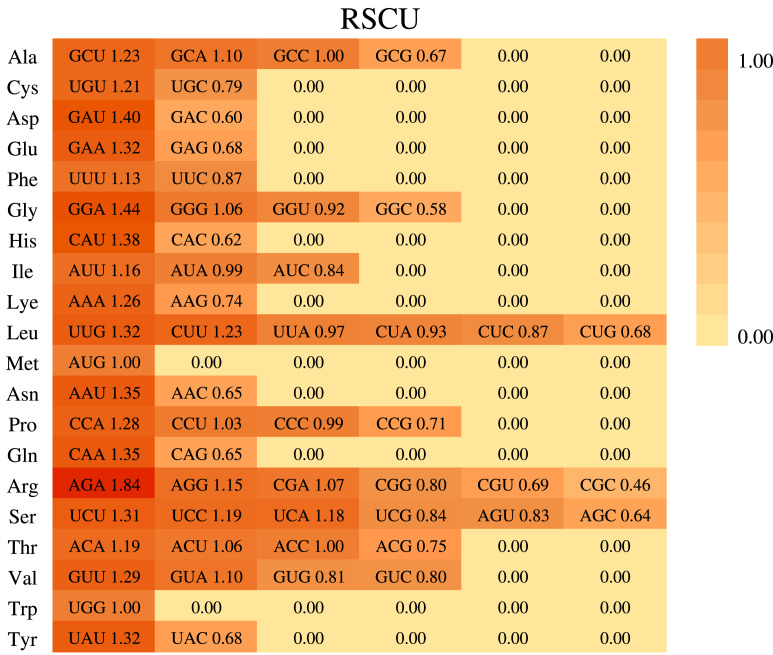
Codon usage in *Phoebe hunanensis*. The codon usage for 20 amino acids as well as stop codons of all protein-coding genes identified is shown for the chloroplast genome of *Phoebe hunanensis*.

Based on the sequences of 84 protein-coding genes from the chloroplast genome of *P. hunanensis*, the codon usage frequency and relative synonymous codon usage (RSCU) were calculated and visualized (Fig. 3). There were 50,930 codons found in the protein-coding genes of the *P. hunanensis* chloroplast genome. Among these codons, the three most abundant ones encode the amino acids leucine (5,242 codons), serine (4,568 codons), and isoleucine (4,170 codons). The three least abundant codons encode tryptophan (740 codons), methionine (971 codons), and cysteine (1,083 codons) (Table S4). Through comparative analysis of codons, we found that AGA had the largest RSCU value of 1.84. Analysis of the 3′-end of codons encoding amino acids revealed that codons with RSCU values >1 usually ended in U or A, and most of those ending in G or C generally had RSCU values <1, such as CGC (arginine), GGC (glycine), and CAC (histidine). In addition, there were four types of codons, GCC, AUG, ACC, and UGG, with RSCU values = 1.

The Ka/Ks values can be used to evaluate whether the coding gene is under selection pressure. We calculated the Ka/Ks values of 79 unique protein-coding genes using *P. neurantha* (NC_039620) as a reference sequence. Ka/Ks values ranged from 0 to 1.3974 (Table S5). The majority of genes had Ka/Ks values below 1. The highest Ka/Ks value was 1.3974 for the *rpl16* gene in *P. tavoyana*. The Ka/Ks values of the *ycf1* and *ycf2* genes were higher than those of other genes, but only the Ka/Ks value of the *ycf2* gene in *P. zhennan* was larger than 1.

### Genome comparison and sequence divergence

**Figure 4:**
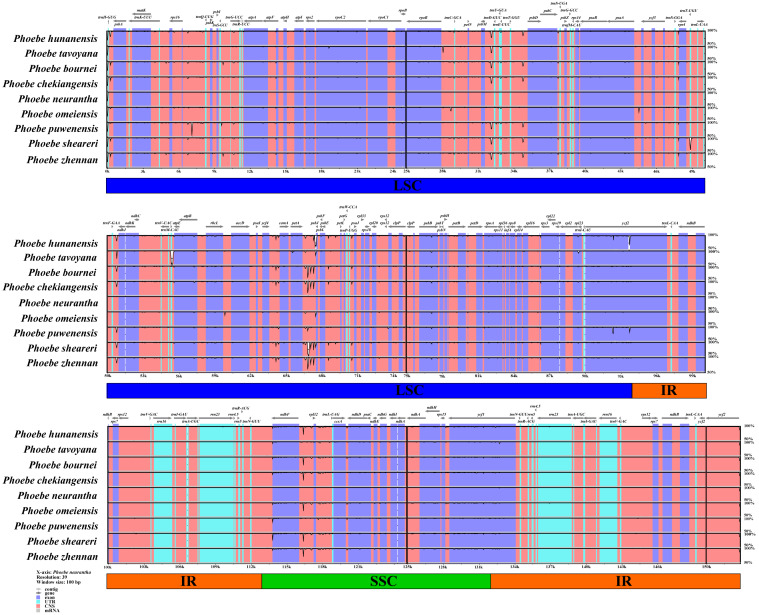
Analysis of the whole chloroplast genomes of *Phoebe* species. The sequence analysis of the *Phoebe* species was performed with mVISTA using *Phoebe neurantha* as the reference sequence, with dark gray arrows indicating the orientation of individual genes, pink bars indicating non-coding sequences (CNS), purple bars indicating exons, blue bars indicating RNA, gray bars indicating mRNA, and the *y*-axis indicating percentage identity, ranging from 50% to 100%.

To determine the level of genomic divergence, sequence identity analysis based on an alignment of the whole chloroplast genomes was performed (Fig. 4). A high degree of conservation was observed among the *Phoebe* chloroplast genomes, and the non-coding regions were more divergent than the coding regions. There were high levels of divergence in the intergenic regions of *psbM–trnD*-GUC, *petA–psbE*, and *ndhF–rpl32*. The coding regions were relatively conserved except for those of *psbL* and *ycf2*. The divergence of chloroplast genome sequences was mainly concentrated in the LSC and SSC regions, while the IR regions had relatively few substitutions.

**Figure 5:**
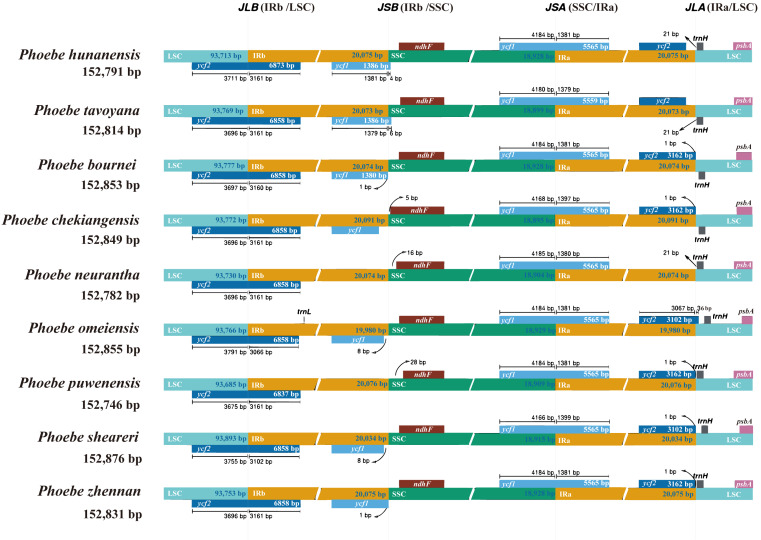
The contraction and expansion of the inverted repeat/single copy (IR/SC) junctions. The boundaries of the IR, short single copy (SSC), and large single copy (LSC) regions in the chloroplast genomes of *Phoebe* species were compared. Loci JLB, JSB, JSA, and JLA represent the LSC/IRb, IRb/SSC, SSC/IRa, and IRa/LSC junctions, respectively.

The genes of the IR/SC junctions in the chloroplast genomes of *Phoebe* species were compared (Fig. 5). JLB, JSB, JSA, and JLA represent the LSC/IRb, IRb/SSC, SSC/IRa, and IRa/LSC junctions, respectively. There were low levels of divergence at each boundary of the genes *ycf2*, *ycf1*, *ndhF*, *trnH*, and *psbA*. *P. neurantha* exhibited one complete *ycf2* copy that crossed the JLB junction, while other *Phoebe* species had two copies, with a truncated pseudogene *ycf2*^*ψ*^ copy at the JLA junction. In *P. neurantha*, the pseudogene *ycf2*^*ψ*^ was missing at the JLA junction. The *ycf1* gene of nine species of *Phoebe* was located at JSA, whereas its corresponding pseudogene *ycf1*^*ψ*^ copy was found at JSB. Notably, this *ycf1*^*ψ*^ pseudogene was missing from *P. neurantha* and *P. puwenensis*. In addition, the *ndhF* gene was located at JSB, contracting by 5 to 28 bp in the SSC region, while the *trnH* gene was situated at JLA, contracting by 21 to 36 bp in the LSC region. The *psbA* gene was contained within the LSC region but not at the end of the LSC region.

### Divergence hotspots in *Phoebe* chloroplast genomes

**Figure 6:**
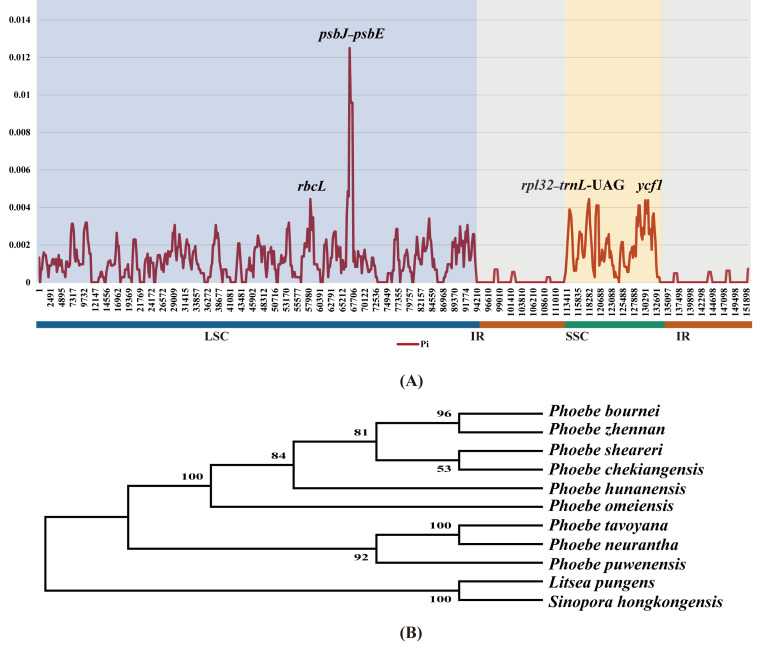
Divergence hotspots. (A) The sliding window analysis of nucleotide diversity (Pi) among the whole chloroplast genomes of nine *Phoebe* species. The *x*-axis represents the base position along the sequences, and the *y*-axis represents the Pi value. (B) A phylogenetic tree using the genes *rbcL* and *ycf1* as well as the intergenic regions *petA–psbE* and *rpl32–trnL*-UAG. These genes and intergenic regions were extracted from the chloroplast genomes of *Phoebe* species.

With the aim of quantifying divergence among the chloroplast genomes of *Phoebe* species at the sequence level, the highly variable regions were analyzed using DnaSP v6 sliding window analysis of nucleotide diversity (Pi) (Fig. 6A). The values of Pi ranged from 0 to 0.0125, and its average was 0.00105. The Pi value in the IR region was extremely low. The Pi value of the SSC region (0.00198) was greater than that of the LSC region (0.00124). In the LSC region, *psbJ–psbE* had the highest Pi value, 0.0125, followed by *rbcL*. In the SSC region, *ycf1* had the highest Pi value. To validate the efficiency of hotspots, we constructed a phylogenetic tree using the genes *rbcL* and *ycf1* as well as the intergenic regions *psbJ–psbE* and *rpl32*–*trnL*-UAG. These genes and intergenic regions were extracted from the chloroplast genomes of nine species of *Phoebe*. The bootstrap values for this phylogenetic tree are all greater than 50 (Fig. 6B).

Single nucleotide polymorphisms (SNPs) in the chloroplast genome were not random, but rather clustered into hotspots. The total number of SNPs ranged from 93 to 228, with a mean value of 185.86 (Table 3). The number of transitions was greater than the number of transversions. There were more A/G transitions than C/T transitions, whereas A/C transversions and G/T transversions predominated among transversions. SNPs were more numerous in the LSC region than in the SSC and IR regions. The numbers of InDels varied from 39 to 71, with a mean value of 60.13 (Table S6). There were more InDels in the LSC region than in the SSC region. There were no InDels in the IR regions, except for *P. puwenensis*. With respect to average InDel lengths, 1.84 bp in the LSC region was also larger than 1.26 bp in the SSC region.

**Table 3:** Comparative analyses of different types of substitutions in *Phoebe* species.

Species	Region	Transition substitutions		Transversion substitutions			
		A/G	C/T	A/T	A/C	C/G	G/T
*Phoebe hunanensis*	Large single copy	44	28	5	30	9	25
*Phoebe tavoyana*		16	11	1	19	2	17
*Phoebe bournei*		44	25	4	31	7	25
*Phoebe chekiangensis*		48	27	3	36	7	21
*Phoebe omeiensis*		12	11	2	15	5	10
*Phoebe puwenensis*		27	23	3	24	5	18
*Phoebe sheareri*		49	25	4	30	8	27
*Phoebe zhennan*		44	25	4	31	7	25
*Phoebe hunanensis*	Inverted repeat	2	1	0	0	0	1
*Phoebe tavoyana*		0	0	0	1	0	0
*Phoebe bournei*		1	1	0	0	0	1
*Phoebe chekiangensis*		1	1	0	0	0	1
*Phoebe omeiensis*		1	1	0	0	0	1
*Phoebe puwenensis*		1	1	0	1	0	0
*Phoebe sheareri*		1	1	0	1	0	2
*Phoebe zhennan*		1	1	0	0	0	1
*Phoebe hunanensis*	Small single copy	20	7	5	6	1	13
*Phoebe tavoyana*		6	2	3	2	0	5
*Phoebe bournei*		19	6	4	6	1	11
*Phoebe chekiangensis*		23	6	4	8	1	13
*Phoebe omeiensis*		20	7	5	6	1	13
*Phoebe puwenensis*		12	5	2	6	1	10
*Phoebe sheareri*		19	7	5	7	1	12
*Phoebe zhennan*		19	6	4	6	1	11

### Phylogenetic analysis

**Figure 7:**
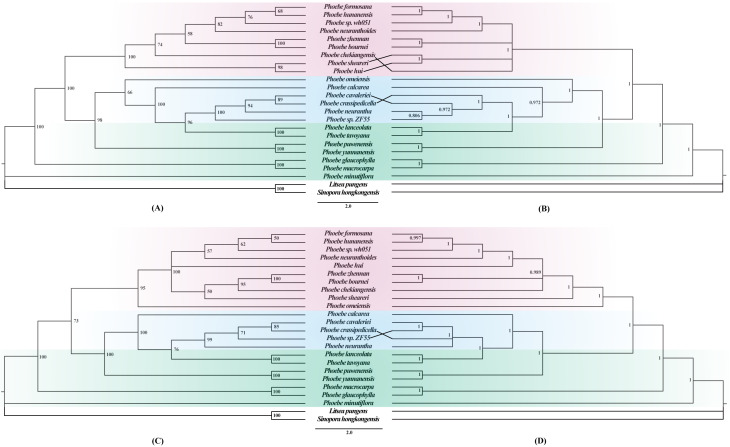
Phylogenetic trees constructed using the maximum likelihood (ML) and Bayesian inference (BI) methods based on the complete chloroplast genomes and protein-coding genes of 22 *Phoebe* species. *Litsea pungens* and *Sinopora hongkongensis* were used as outgroups. Support values are indicated by the numbers above the nodes, ML bootstrap values are indicated by the numbers in (A) and (C), and Bayesian posterior probability (PP) values are indicated by the numbers in (B) and (D).

To better understand the phylogenetic relationships among different *Phoebe* species, phylogenetic trees were constructed based on whole chloroplast genome sequences and a separate dataset of 79 protein-coding genes using maximum likelihood (ML) and Bayesian inference (BI) approaches. ML and BI trees of complete chloroplast genomes and protein-coding genes exhibited similar topologies (Fig. 7). The support values for each branch node in the four phylogenetic trees were also high. In the phylogenetic trees based on both the chloroplast genome (Figs. 7A and 7B) and the protein-coding genes (Figs. 7C and 7D), the *Phoebe* species were grouped into three branches, with *P. minutiflora*, *P. glaucophylla*, and *P. macrocarpa* forming a monophyletic basal clade. There were some slight differences between the four phylogenetic trees produced from the complete chloroplast genome sequences and protein-coding region sequences, with the most notable one being the relationship of *P. omeiensis* to other *Phoebe* species. Furthermore, the position of *P. hui* varied in different phylogenetic trees. Both trees indicated that *P. tavoyana* was most closely related to *P. lanceolata* and that *P. hunanensis* was most closely related to *P. formosana*. In these phylogenetic trees, almost all *Phoebe* species from Yunnan form the basal group. *Phoebe* species from southwestern China were clustered together into one branch, while *Phoebe* species from central and southeastern China were clustered together into another branch (Fig. 7).

### Phylogeographical analysis of *Phoebe* species in China

**Figure 8:**
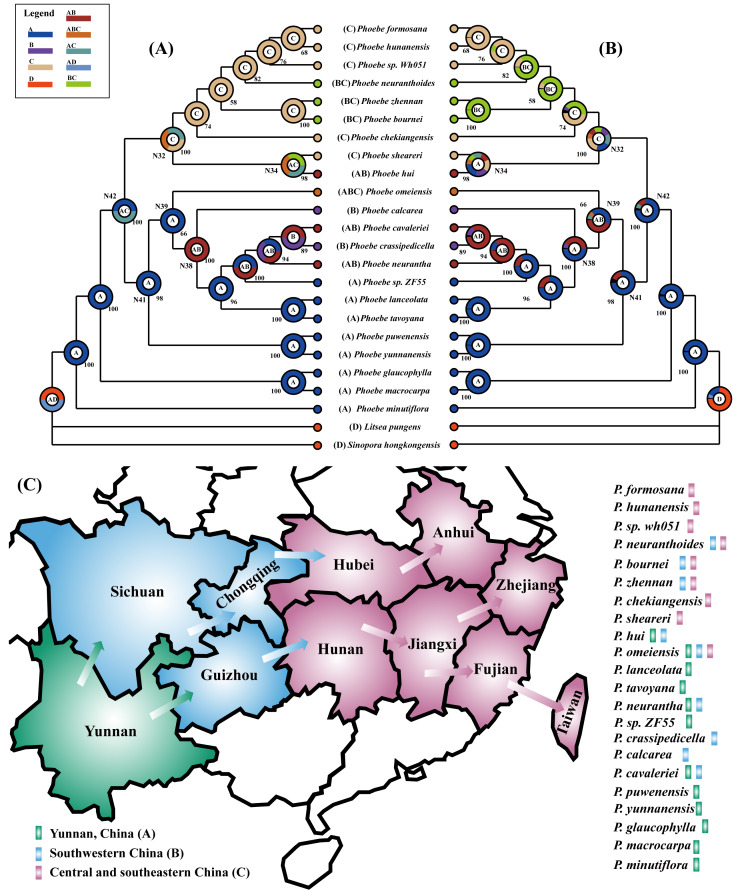
Phylogeographical analysis of *Phoebe* species in China. Using the ancestor reconstruction software RASP to forecast the origin and dispersal of *Phoebe* species in China utilizing S-DIVA (Statistical Dispersal-Vicariance Analysis) and BBM (Bayesian Binary MCMC) methodologies. The letters A, B, C, and D in the legend at the upper left stand for Yunnan, southwestern China, central and southeastern China, and distribution of outgroups, respectively. The combination of these distribution sites is represented by a string of letters. The letter inside the circle represents the ancestral distribution with the highest probability of branching at that node. (A) Results of ancestral distribution area reconstruction using the S-DIVA method. (B) Results of ancestral distribution area reconstruction using the BBM method. (C) Diagram of the origin and dispersal of *Phoebe* species in China. Green represents Yunnan Province, China, blue represents southwestern China, and pink represents central and southeastern China. The arrows represent the direction of dispersal. The different colors of the rectangle after the species name represent the geographical distribution of each *Phoebe* species.

The four phylogenetic trees indicated a clear pattern in the origin and dispersal of these *Phoebe* species. To further validate the pattern, we used the ancestor reconstruction software RASP to forecast the origin and dispersal of *Phoebe* species in China utilizing S-DIVA and BBM methodologies. The 22 *Phoebe* species in China were grouped into three geographic regions according to their distributional ranges: (A) Yunnan, China; (B) southwestern China; (C) central and southeastern China. As it was not possible to determine the suitability of the distribution of the outgroup, we set it to D. The results of the S-DIVA and BBM ancestral reconstructions were similar (Figs. 8A and 8B). The results of both analytical methods broadly divide the 22 *Phoebe* species in China into two clades. Ancestors of one clade were primarily concentrated in Yunnan, whereas those of the other were located in central and southeastern China. Several nodes, including 32, 34, 38, 39, 41, and 42, had multiple ancestral distributions that varied across the two analytic approaches (Table S7). The ancestral components of nodes 32 and 34 were complex. The highest ancestral probability at node 32 of both analysis methods was the C distribution type. In contrast, the A distribution type was highest in the BBM analysis at node 34, while the AC distribution type was highest in the S-DIVA analysis. Based on the complete chloroplast genome of *Phoebe* species in China, S-DIVA and BBM analyses revealed that Yunnan was the ancestral region of the basal taxa of *Phoebe* species. Thus, it can be reasonably inferred that Yunnan is the origin of the genus *Phoebe*, from which species in southwestern China and southeastern China, respectively, diversified (Fig. 8C).

## Discussion

### Characterization of chloroplast genomes in *Phoebe*

In this study, we first performed a comparative analysis of *Phoebe* chloroplast genomes. No obvious differences were found among the structures and sequences of these *Phoebe* chloroplast genomes. The chloroplast genome size of nine species of *Phoebe* measured 152,746–152,876 bp, similar to those of other species in the family Lauraceae (Song et al., 2018; Zhao et al., 2018; Ge et al., 2019). The percentage of chloroplast genome content and gene order in *Phoebe* species were similar to those previously reported in angiosperms (Tian et al., 2018; Zhang et al., 2021). In comparison with AT base pairs, GC base pairs are more thermodynamically stable (Wang et al., 1984), whereby the influence of GC content on chloroplast genome stability is greater (Yang et al., 2022). The chloroplast genomes of *Phoebe* species had a total GC content of 39.1%, consistent with the genomes of *Alseodaphne*, *Cinnamomum*, and *Litsea* (Chen et al., 2017; Hinsinger & Strijk, 2017; Song et al., 2018). The similar GC content has apparently ensured the stability of the chloroplast genomes in Lauraceae species. SSRs were widely present in the chloroplast genomes, generally in the form of tandem repeats with 1–6 nucleotide motifs (Kim, Lee & Choi, 2021). In the present study, the quantity of SSRs ranged from 209 to 215, with mononucleotide repeats being the most abundant. The mononucleotide SSRs were significantly abundant in A/T repeats. The SSRs of the chloroplast genomes of *Phoebe* species generally consisted of thymine or adenine repeats, while cytosine and guanine repeats were rare, which was consistent with a previous study of Lauraceae (Chen et al., 2017). Repeat sequences have been mentioned in previous studies as playing a vital role in the rearrangement, stabilization, and phylogenetic analysis of chloroplast genomes (Cavalier-Smith, 2002; Nie et al., 2012). The palindromic and forward repeats were the dominant repeat types in the *Phoebe* chloroplast genome, suggesting that palindromic and forward repeats were more favorable to the regulation of gene expression during the evolution of *Phoebe* species (Koller & Delius, 1982). Similar to previous studies, the long repeat sequences were mainly positioned in the intergenic spacer and intron regions (Ge et al., 2019; Abdullah Mehmood et al., 2020). The LSC region has been previously determined to contain a large number of intergenic sequences (Ruang-Areerate et al., 2021), and there were a large number of repetitive sequences in the LSC region compared to the IR and SSC regions in the present study. The amino acids in an organism can be encoded by two or more codons in general owing to codon degeneracy (Zuo et al., 2017). Codon bias and the accumulation of substitutions that optimize codon use have played a significant role in the evolution of the chloroplast genome (Shahzadi et al., 2020). RSCU is the ratio of the frequency a synonymous codon has been used to the frequency that was expected, which can be used as a meaningful indicator of codon bias (Mo et al., 2020). Among all codons, AGA had the highest RSCU value (1.84), and the high RSCU value could be related to the function of the arginine being encoded by AGA, which could be associated with avoiding transcriptional errors (Gao et al., 2019). We performed an estimation of evolutionary rates for 79 unique protein-coding genes. The results indicated that most genes were selected by purification. Only two genes, the *ycf2* gene in *P. zhennan* and the *rpl16* gene in *P. tavoyana*, were under positive selection. Based on earlier evolutionary assessments of *Litsea* species (Song et al., 2022b) as well as *Phoebe* species here, the majority of the coding genes in Lauraceae species remained stable during the evolution process, except for *ycf2* and *rpl16* genes. The comparative analysis revealed that the chloroplast genomes of *Phoebe* were relatively conserved, which could practically inform future studies on Lauraceae species.

### Chloroplast genomic divergence among *Phoebe* species

Although the chloroplast genomes were relatively conserved, divergence was still observed. The mVISTA results indicated a high degree of sequence similarity among the chloroplast genomes of *Phoebe* species, which was similar to results in other Lauraceae studies (Ge et al., 2019; Wang et al., 2021b). The divergence among chloroplast genome sequences was mainly concentrated in the LSC and SSC regions, with relatively low diversity in the IR regions. The non-coding regions were more divergent than the coding regions. These phenomena were also identified in angiosperms more broadly (Yao et al., 2015; Zhang et al., 2016). In the intergenic regions, the *psbM–trnD*-GUC, *petA–psbE*, and *ndhF–rpl32* regions were highly divergent, while the coding regions were relatively conserved except within *ycf2*, which could thus be used for species identification. Although the genes in the IR regions are highly conserved (Shen et al., 2017), it is very common for the IR/SC boundaries to contract and expand (Terakami et al., 2012; Yang et al., 2016). Contraction and expansion of the IR/SC boundaries can promote the divergence of genes in the chloroplast genome, leading to gene deletion and pseudogene creation, which can be used in phylogenetic analysis (Li et al., 2019). Pseudogenes and gene deletions emerged at JLA and JSB in the *Phoebe* species, according to analysis of genes surrounding the IR/SC boundaries in the chloroplast genomes. No variation was observed at JLB and JSA. This result suggested that JLA has evolved through expansion and contraction, whereas JLB has been far more conserved (Vaillancourt & Jackson, 2000). At JLB, the pseudogene *ycf2*^*ψ*^ of *P. neurantha* was lost. The *ycf1* gene was located at JSA, whereas its corresponding pseudogene *ycf1*^*ψ*^ copy was found at JSB. Notably, this pseudogene *ycf1*^*ψ*^ copy was missing from *P. neurantha* and *P. puwenensis*, which agrees with previous studies showing that the *ycf2* and *ycf1* genes in the chloroplast genome were associated with the divergence of the IR/SC boundaries and tended to be pseudogenes (Huang, Chen & Li, 2017; Gu et al., 2018; Ge et al., 2019). The detection of highly variable regions and the calculation of nucleotide diversity could quantify divergence in the chloroplast genome at the sequence level (Li et al., 2020). The *petA*–*psbE* and *rpl32*–*trnL*-UAG regions and *rbcL* and *ycf1* were determined to have high Pi values. These regions could be experiencing accelerated nucleotide substitutions at the species level, which demonstrates their potential as molecular markers with applications in plant identification and phylogenetic analysis (Suo et al., 2016). In addition, to validate the efficiency of hotspots, we extracted the corresponding sequences to construct a phylogenetic tree. The phylogenetic results supported the prior phylogenetic study using complete chloroplast genomes (Zhang, Li & Wang, 2020), indicating that corresponding sequences are available for phylogenetic analysis. The study of structural variation in the chloroplast genome was important for the study of species evolution (Sebbenn et al., 2019). The SNPs and InDels were shown to be more informative than chloroplast DNA fragments in the inference of species delimitation and population studies at different taxonomic levels (Song et al., 2017a; Zhou et al., 2020). The results of analyses of SNPs and InDels suggested that microstructural mutations in the IR regions were very rare, as demonstrated in previous studies (Yang et al., 2016; Li et al., 2017).

### Phylogenetic analysis and biogeographic origin of *Phoebe* species in China

Over the years, the chloroplast genome played an important role in the analysis of phylogenetic relationships among closely related species and in illustrating patterns of evolution among angiosperms (Jansen et al., 2007; Kim et al., 2015). So far, phylogenetic analyses of the genus *Phoebe* have been based only on highly variable markers (Song et al., 2017a). To obtain a more reliable result, several studies have constructed phylogenetic trees using complete chloroplast genome sequences and protein-coding regions and compared them to analyze the phylogenetic relationships among species (Sheng et al., 2021; Song et al., 2022c). In the present study, *Phoebe* species were all divided into three branches in the phylogenetic trees based on their chloroplast genomes and protein-coding genes. Phylogenetic analysis revealed that *P. tavoyana* belonged to the basic clade and *P. hunanensis* was in the apical clade. The outcomes provided by the ML and BI trees differed little as well. However, there were some slight differences between the phylogenetic tree of complete chloroplast genomes and phylogenetic tree of protein-coding genes. *P. omeiensis* was distributed among different sub-branches in the trees. *P. omeiensis* was sister to *P. neurantha* in a previous study (Zhang et al., 2021) that constructed a phylogenetic tree based on complete chloroplast genomes, while *P. omeiensis* was sister to *P. sheareri* in another study (Ge et al., 2019) which constructed a phylogenetic tree using protein-coding genes. Comparatively, we discovered that both the ML tree and the BI tree had greater support values for the phylogenetic tree constructed from the complete chloroplast genome. Such different trees could originate from substitutions in the intergenic spacer regions, which illustrates the importance of non-coding regions in the phylogenetic analysis. The 22 *Phoebe* species found in China have been divided into three clades for the first time using a phylogenetic tree built utilizing the complete chloroplast genome and protein-coding genes. The study of genetic diversity is essential for biodiversity conservation (Cruz et al., 2012).

We found a pattern regarding the phylogenetic analysis of *Phoebe* species endemic to China in the three branches. All *Phoebe* species in the basal branch are distributed in Yunnan Province, China. *Phoebe* species within another branch are widespread in southwestern China, while *Phoebe* species in the other branch are spread across central and southeastern China. To further validate the pattern, we used RASP to carry out a phylogeographical analysis of the *Phoebe* species in China. Based on the complete chloroplast genome of *Phoebe* species in China, S-DIVA and BBM analyses showed that Yunnan is the center of origin and divergence of *Phoebe* species in China. We have developed assumptions about the origin and dispersal routes of *Phoebe* species in China based on the RASP results. We infer that the *Phoebe* species in China originated in Yunnan, which is known as a cradle of plant diversity (Qian et al., 2020). Subsequently, *Phoebe* species spread from Yunnan to southwestern China. Finally, *Phoebe* species spread from southwest China to central and southeastern China. Environmental variation has played a significant role in speciation and species diversity (Schluter & Pennell, 2017). Our results have indicated that geographical isolation has resulted in genetic diversity among *Phoebe* species. The findings of this study serve as a foundation for further investigation into the origin and evolution of the diversity of *Phoebe* species in China, which should be taken into account in future biodiversity conservation plans.

## Conclusions

In this study, we conducted the first comprehensive comparative analysis of the chloroplast genomes of diverse *Phoebe* species. We characterized chloroplast genomes of various *Phoebe* species in China. Despite the conserved nature of chloroplast genomes, we detected some highly divergent intergenic regions (*petA*–*psbE*, *ndhF*–*rpl32*, and *psbM*–*trnD*-GUC) as well as three highly divergent genes (*rbcL*, *ycf1*, and *ycf2*) that have potential applications in phylogenetics and evolutionary analysis. In addition, the *ycf1* and *ycf2* genes in *Phoebe* species were associated with the divergence of the IR/SC boundaries and tended to be pseudogenes or missing. The results of phylogenetic analysis indicated that various *Phoebe* species in China were divided into three clades. The complete chloroplast genome was better suited for phylogenetic analysis of *Phoebe* species. Based on phylogeographical analysis of *Phoebe* species in China, we infer that the *Phoebe* species in China have their evolutionary origin in Yunnan. The results of this study should be utilized in future conservation plans for *Phoebe* species diversity and studies of the evolution of the genus.

##  Supplemental Information

10.7717/peerj.14573/supp-1 Supplemental Information 1 Information of species in phylogenetic tree

10.7717/peerj.14573/supp-2 Supplemental Information 2 Statistics of SSRs

10.7717/peerj.14573/supp-3 Supplemental Information 3 Statistics of long repeats

10.7717/peerj.14573/supp-4 Supplemental Information 4 Codon usage details of *Phoebe hunanensis*

10.7717/peerj.14573/supp-5 Supplemental Information 5 Statistics of the Ka/Ks values for the various Phoebe species’ protein-coding genes

10.7717/peerj.14573/supp-6 Supplemental Information 6 InDels statistics

10.7717/peerj.14573/supp-7 Supplemental Information 7 Probabilities of ancestral distribution areas for significant nodes

10.7717/peerj.14573/supp-8 Supplemental Information 8 The aligned complete chloroplast genome and protein-coding gene sequences in this study

## Additional Information and Declarations

### Data Availability

The following information was supplied regarding data availability: The data that support the findings of this study are available at GenBank: MZ442606–MZ442607.
